# Monitoring the Subolesin Vaccine Field Trial for Safer Control of Cattle Ticks Amidst Increasing Acaricide Resistance in Uganda

**DOI:** 10.3390/vaccines10101594

**Published:** 2022-09-22

**Authors:** Fredrick Kabi, Moses Dhikusooka, Moses Matovu, Swidiq Mugerwa, Paul Kasaija, Patrick Emudong, Halid Kirunda, Marinela Contreras, Christian Gortazar, Jose De la Fuente

**Affiliations:** 1 National Livestock Resources Research Institute (NaLIRRI/NARO), Kampala P.O. Box 5704, Uganda; 2 SaBio, Instituto de Investigación en Recursos Cinegéticos (IREC), Consejo Superior de Investigaciones Cientí ficas (CSIC), Universidad de Castilla-La Mancha (UCLM)-Junta de Comunidades de Castilla-La Mancha (JCCM), Ronda de Toledo 12, 13005 Ciudad Real, Spain; 3 Mbarara Zonal Agricultural Research and Development Institute (MbaZARDI), Mbarara City P.O. Box 389, Uganda; 4 Department of Veterinary Pathobiology, Center for Veterinary Health Sciences, Oklahoma State University, Stillwater, OK 74078, USA

**Keywords:** Subolesin, vaccine, cattle, tick, field trial

## Abstract

A collaboration program was established between the group of Health and Biotechnology (SaBio) of the IREC Institute of Game and Wildlife Research (CSIC-UCLM-JCCM, Spain) and the National Agricultural Research Organization of Uganda (NARO) for the development of vaccines for the control of cattle ticks in Uganda. Controlled pen trials identified a tick protective antigen, *Rhipicephalus appendiculatus* Subolesin, and a cross-species-effective vaccine formulation. As the next step, a controlled vaccine field trial has been approved by Ugandan state regulatory authorities, the Uganda National Council for Science and Technology (UNCST) and the National Drug Authority (NDA), to evaluate the efficacy and effectiveness of the vaccine formulation for the control of cattle tick infestations under field conditions. The results of this trial may lead to the approval of the vaccine for application in Uganda to improve cattle health and production while reducing the use of acaricides.

## 1. Introduction

Ticks parasitize livestock and wild animals with a higher prevalence in the warm tropics and sub-tropics while humans are accidental hosts, with all at risk for tick-borne diseases [1]. Economic losses due to tick infestations range from USD 22 to 30 billion annually worldwide [1]. Furthermore, tick-suitable habitats keep widening due to human activities and climatic changes associated with increasing global warmth.

In Uganda, the most common tick species are the brown ear ticks (*Rhipicephalus appendiculatus*), blue ticks (*R. decoloratus*), bont ticks (*Amblyomma variegatum*), and red ticks (*R. evertsi*) [2,3,4,5,6]. Ticks are responsible for USD 1.1 billion in annual losses, resulting from livestock deaths due to tick-borne pathogens, related morbidity, costly but unreliable treatment of tick-borne diseases, acaricide resistance, blood loss from hosts, and tick-associated tissue damages, among other factors [3]. As stated by cattle farmers, if cattle are not sprayed with a potent acaricide for two weeks, tick infestation varies between 30–45 ticks per animal. The three-host brown ear tick, *R. appendiculatus*, is the most common tick infesting cattle in Uganda and other southern and southeastern African countries [2,6]. As observed in the field, it is common to find hares acting as reservoir hosts on cattle farms adhering to strict tick control. The *R. appendiculatus* lifecycle can be completed in three months, thus able to have several cycles per year. This tick transmits *Theileria parva*, the cause of a febrile and most lethal cattle tick-borne disease, East Coast fever (ECF), and Corridor disease in southern Africa. The nymph and adults have the capacity to acquire and transmit infection. It has been speculated that *R. appendiculatus* has been introduced to South Sudan from Uganda through the export of infested cattle. The second most common tick is the one-host tick, *R. decoloratus*, whose main host is cattle, and its preferred sites of attachment include the shoulders, dewlap, neck, and belly [2,4]. All the developmental stages spend about three weeks on the same host, and its life cycle is about 60 days, inclusive of the non-parasitic phases, thus having more than one life cycle annually in Uganda. The *R. decoloratus* ticks occur in savanna and temperate climates, naturally in grasslands and wooded areas used as cattle pastures. The third most prevalent tick in Uganda is a three-host tick, *A. variegatum*, which infests cattle, sheep, and goats. Adults’ preferred attachment sites include the dewlap, sternum, flanks, genitalia and their surroundings, and the udders. This tick is most abundant in the rainy season, has only one life cycle per annum, and transmits *Cowdria ruminantium*, which causes heartwater. This tick is distributed through west, central, and east Africa, and in southern Africa, it extends into Zambia, northeastern Botswana, the Caprivi Strip of Namibia, northwestern Zimbabwe, and central and northern Mozambique. The red-legged tick, *R. evertsi*, is less common in Uganda but is commonly encountered infesting cattle and donkeys which are reared together in the northeastern region [2]. This tick is a medium-sized, two-host tick that is dark brown with reddish-orange legs. This tick can undergo more than two lifecycles per annum and transmit *Anaplasma marginale* for cattle and *Babesia caballi* for donkeys [2,5].

For over a century, the control of ticks has mainly relied on acaricides globally. However, over time, acaricide usefulness has been overwhelmed by prolonged use and associated misuse culminating in a selection of acaricide-resistant tick populations. This scenario demands the research and deployment of safer control approaches such as anti-tick vaccines to curtail the currently escalating costs of cattle farming and environmental and food contamination [2]. However, only two vaccines, TickGard and Gavac, have been registered and commercialized [6].

Since 2014, scientists based at the National Livestock Resources Research Institute (NaLIRRI) under the National Agricultural Research Organisation (NARO) in Uganda in collaboration with Health and Biotechnology (SaBio), IREC Institute of Game and Wildlife Research (CSIC-UCLM-JCCM) in Spain have been engaged in research to develop Subolesin-based vaccines for the control of ticks and tick-borne diseases in cattle. Since then, research has progressed through the proof of concept and on-station clinical trial stages to identify *R. appendiculatus* Subolesin as a tick-protective antigen for a cross-species effective vaccine formulation [7]. These studies, mainly funded by the government of Uganda, were initiated to avert the widespread occurrence of tick acaricide resistance [8]. According to the National Drug Authority of Uganda, the use of a given acaricide should be changed every 24 months or earlier to avoid the selection of acaricide-resistant ticks.

## 2. Subolesin Vaccine Field Trial

Presently, plans have advanced to conduct controlled field anti-tick vaccine trials having been approved by state regulatory authorities to inform prospectus licensure for vaccine commercialization. This is the first field trial in Uganda and an opportunity for researchers, cattle farmers, and other value chain actors in the cattle industry to benefit from a novel candidate vaccine for tick control.

The trial will determine the candidate Subolesin-based anti-tick vaccine safety, efficacy, and effectiveness among cattle (minimum 330 animals) distributed proportionately in five farms with known tick and tick-borne disease challenges. This sample size was calculated using the formula previously developed [9,10,11] while adhering to the principle of three Rs, replacement, reduction, and refinement [12], and in line with the national guidelines for the use of animals for research and teaching. The trial adopts a multi-site, double-blinded, randomized, controlled field trial, with two group comparisons per site, vaccinated versus a placebo adjuvant-alone treated per herd. The trial will span for a period of 365 days to enable the determination of the annual booster dose timing, and any peculiar side effects which could be associated with the vaccine.

**Figure 1:**
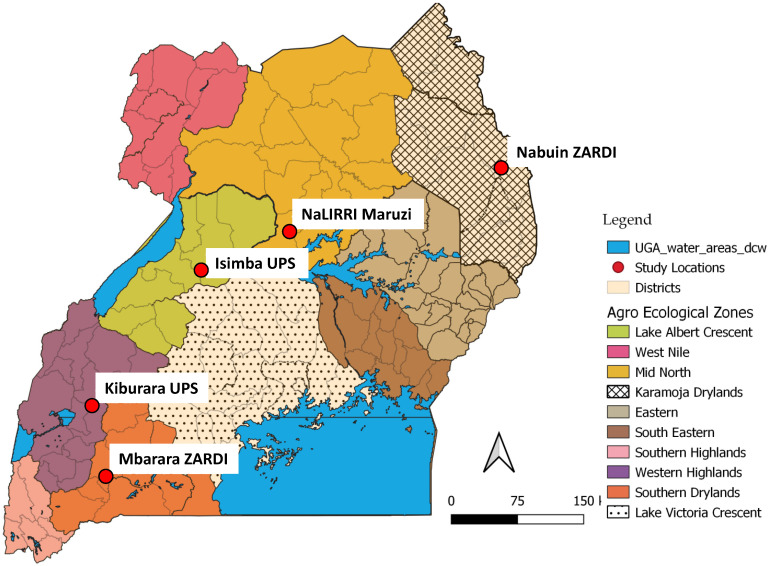
Uganda agroecological zones with locations of the trial coordination center and experimental cattle sites.

During tick peak seasons, cattle losses are due to tick-borne diseases such as ECF, anaplasmosis, babesiosis, and heartwater. The five trial farms owned by NARO and the Uganda government prison services are each based in different agroecological zones in northeastern semi-arid, mid-northern savannah grasslands, Lake Albert crescent, western highlands, and southwestern rangelands (Figure 1). The distribution of the trial sites takes into consideration several variables including the common cattle breeds composition, multiple tick species challenge, diversity, and agroecology in Uganda and the experimental region. The selected trial cattle farms use acaricides for tick control with an application frequency of two to three times per week. Previously, these sites used a combination acaricide of Chlorpyrifos 50% and Cypermethrin 5% (*w*/*v*), while the Nabuin site (north-eastern semi-arid site) employed a single compound, Cypermethrin 10%. Before the trial starts, all the experimental farms will use a combination acaricide Chlorpyrifos 50% and Cypermethrin 5% (*w*/*v*), marketed as Duodip^®^ in Uganda. This was standardized to avoid trial biases associated with acaricide use in an integrated control strategy to prevent acaracide use after vaccination. Common cattle breeds belonging to the East African Shorthorn Zebu, Longhorn Ankole, Boran, and crossbreed Friesians have been included in the trial. These cattle experimental sites are located within the “cattle corridor” where cattle provide the main source of livelihood.

The study design considers the random selection of experimental cattle and the maintenance of blinding the candidate vaccine and control treatment, collecting and analyzing data to avoid experimental biases, having blinded vaccinated and control cattle groups (identified with different colored ear tags) at each experimental site to enhance a similar exposure to natural tick infestations, and maintaining experimental cattle welfare with reference to freedom from disease and pain, thirst and hunger, discomfort due to inadequate space, distress and fear, and denial to engage in natural behavior. The study will evaluate specific Subolesin antibody titers and weights of feeding ticks to determine the differences between ticks collected from vaccinated and control cattle. To achieve this goal, the following steps will be undertaken:

Half-body tick counts will be conducted before each immunization and twice a month from ten cattle at each experimental farm ensuring that five animals from the vaccinated and adjuvant-alone treated cattle are included. The ticks will be morphologically classified at species levels. This will generate data on the differences in tick infestations.

Subolesin antibody titers will be determined by the collection of blood samples every 7 days after carrying out the half-body tick counts. Sera will be obtained, and IgG antibody titers will be determined using an indirect antigen-specific ELISA [7]. The antibody titers will enable the correlation of tick infestation dynamics and host immunity in response to the vaccine.

Five engorged female ticks for each identified species will be selected from each sampled cattle in step (a) and kept separately in well-labeled ventilated bottles. Ticks will be weighed with precision to determine the differences in weights associated with feeding on the vaccine- and adjuvant-treated cattle.

The weighed ticks from step (b) will then be incubated at an appropriate temperature and humidity to enable oviposition, and the egg weights will be determined to establish differences in oviposition.

The egg mass from step (c) will be incubated to enable hatching. This will determine differences in egg fertility.

With all the above considerations, the study will be able to determine the candidate vaccine efficacy for each tick species using the formula outlined below as previously used (e.g., [13]). The following steps will be followed:

The candidate vaccine effect on the number of adult female ticks (DT) = 100[1 − (NTV/NTC)], where NTV is the number of adult female ticks in the vaccine-treated group, and NTC is the number of adult female ticks in the control (adjuvant-only-treated) group.

The candidate vaccine effect on the tick weight (DW) = 100[1 − (WTV/WTC)], where WTV is the average adult female tick weight in the vaccinated group, and WTC is the average adult female tick weight in the control (adjuvant-only-treated) group.

The candidate vaccine effect on oviposition (DO) = 100[1 − (PATV/PATC)], where PATV is the average weight of the eggs per survived tick in the vaccine-treated group, and PATC is the average weight of the eggs per survived tick in the control (adjuvant-only-treated) group.

The candidate vaccine effect on egg fertility (DF) = 100[1 − (PPLOV/PPLOC)], where PPLOV is the average weight of the larvae per gram of eggs in the vaccinated group, and PPLOC is the average weight of the larvae per gram of eggs in the control group.

Finally, calculate the vaccine efficacy (E) = 100[1 − (CRT × CRO × CRF)], where CRT = NTV/NTC, CRO = PATV/PATC, and CRF = PPLOV/PPLOC, which is a representation of the reduction in the number of adult female ticks, oviposition, and egg fertility as compared to the control group, respectively.

## 3. Conclusions

In conclusion, if vaccine efficacy and effectiveness are supported by the results of the trial, Ugandan state regulatory authorities will consider the approval of the vaccine for prospectus licensure for vaccine commercialization. These results will represent a key step for the Ugandan cattle industry with a positive impact on society and the economy.

## Data Availability

Not applicable.
